# Systematic Characterization of MicroRNA Processing Modes in Plants With Parallel Amplification of RNA Ends

**DOI:** 10.3389/fpls.2021.793549

**Published:** 2021-12-07

**Authors:** Ning Li, Guodong Ren

**Affiliations:** State Key Laboratory of Genetic Engineering and Ministry of Education Key Laboratory for Biodiversity Science and Ecological Engineering, School of Life Sciences, Institute of Plant Biology, Fudan University, Shanghai, China

**Keywords:** microRNA, plants, degradome, parallel analysis of RNA ends (PARE), DCL1

## Abstract

In plants, the RNase III-type enzyme Dicer-like 1 (DCL1) processes most microRNAs (miRNAs) from their primary transcripts called pri-miRNAs. Four distinct processing modes (i.e., short base to loop, sequential base to loop, short loop to base, and sequential loop to base) have been characterized in Arabidopsis, mainly by the Specific Parallel Amplification of RNA Ends (SPARE) approach. However, SPARE is a targeted cloning method which requires optimization of cloning efficiency and specificity for each target. PARE (Parallel Amplification of RNA Ends) is an untargeted method *per se* and is widely used to identify miRNA mediated target slicing events. A major concern with PARE in characterizing miRNA processing modes is the potential contamination of mature miRNAs. Here, we provide a method to estimate miRNA contamination levels and showed that most publicly available PARE libraries have negligible miRNA contamination. Both the numbers and processing modes detected by PARE were similar to those identified by SPARE in Arabidopsis. PARE also determined the processing modes of 36 Arabidopsis miRNAs that were unexplored by SPARE, suggesting that it can complement the SPARE approach. Using publicly available PARE datasets, we identified the processing modes of 36, 91, 90, and 54 miRNAs in maize, rice, soybean, and tomato, respectively, and demonstrated that the processing mode was conserved overall within each miRNA family. Through its power of tracking miRNA processing remnants, PARE also facilitated miRNA characterization and annotation.

## Introduction

MiRNAs are a class of endogenous small non-coding RNAs that direct post-transcriptional gene silencing through bases complementary with their target genes. Most miRNAs are transcribed by DNA-dependent RNA polymerase II (Pol II); they are folded into imperfect stem-loop structures, and undergo processing by the RNase III enzyme-containing microprocessor (Song et al., 2019; Wang et al., 2019). Precise releasing of miRNA/miRNA* duplexes from their precursor RNAs is vital for miRNA biogenesis with minimal off targets. Understanding the molecular features of miRNA processing is beneficial for designing artificial miRNAs with enhanced efficiency.

In animals, pri-miRNAs exhibit rigid secondary structures that consist of a ∼35-bp stem, a terminal loop, and long single-stranded RNAs (ssRNAs) flanking the fold-back region (Han et al., 2006). Pri-miRNAs are diced by Drosha at the lower stem ∼11-bp away from the ssRNA-dsRNA junction, releasing pre-miRNAs with 2 nucleotides (nt) overhangs (Han et al., 2006; Kwon et al., 2016). After export to the cytoplasm, a second cut is performed by Dicer at ∼22-bp upstream of the Drosha cut site, yielding mature miRNA-5p/miRNA-3p (miR-5p/miR-3p) duplexes (Grishok et al., 2001; Ketting et al., 2001; Treiber et al., 2019). In plants, however, pri-miRNAs are more heterogeneous in size, with fold-back lengths varying from 60 to over 500 nt (Reinhart et al., 2002; Zhang et al., 2006; Cuperus et al., 2011). Consequently, four different processing modes have been described: (a) short base to loop; (b) short loop to base; (c) sequential base to loop; and (d) sequential loop to base (Bologna and Voinnet, 2014). In the canonical short base-to-loop mode, an internal bulge at 15–17 bp below the miR-5p/miR-3p region guides the first cut at the loop-distal miRNA site (Cuperus et al., 2010; Mateos et al., 2010; Bologna et al., 2013; Moro et al., 2018). Whereas in the loop-to-base mode, a terminal loop or bulge at 15–17 bp above the miR-5p/miR-3p region directs DCL1 processing from the loop-proximal cleavage site (Bologna et al., 2009, 2013; Axtell et al., 2011; Moro et al., 2018). In both cases, the 15–17 bp lower or upper stem tends to be conserved with paired status at different taxonomic levels (Chorostecki et al., 2017). Longer stems may cause additional cuts, which are termed sequential base-to-loop or loop-to-base processing modes, according to their processing direction.

Specific Parallel Amplification of RNA Ends (SPARE) is a modified 5′ RACE (rapid amplification of cDNA ends) with pri-miRNA specific primers for targeted cloning of 3′ DCL processing remnants. It provides a high-throughput solution for the identification of miRNA processing modes (Bologna et al., 2013). Although the method provides high precision and sensitivity, it only clones DCL processing remnants with predesigned primers. Parallel Amplification of RNA Ends [PARE; also referred to as degradome sequencing and GMUCT (genome-wide mapping of uncapped transcripts)] is a well-established method that captures 5′ termini of uncapped and polyadenylated RNA fragments. It has been widely used to track miRNA mediated target slicing events and to estimate RNA degradation levels (Addo-Quaye et al., 2008; German et al., 2008; Gregory et al., 2008). In principle, PARE could also detect 3′ dicing products and infer miRNA processing modes in an untargeted manner. In fact, it has been employed to discover the conserved loop-first processing of miR319 precursors in plants (Addo-Quaye et al., 2009). Concern over mature miRNAs contamination limits its use in probing miRNA processing (Lu et al., 2009). However, there have been no rigorously tests to determine whether PARE tags matching mature miRNA sequences are a result of contamination or not.

Using SPARE results in Arabidopsis as a benchmark, we here provided a solution for the estimation of mature miRNA contamination of PARE data. We found that most PARE tags matching mature miRNA sequences were likely not miRNA contaminations. Overall, PARE had comparable accuracy and sensitivity to SPARE and could be used as a complementary method. Using publicly available PARE data, we systematically identified miRNA processing modes in four crop species and found that miRNAs within the same family tended to share the same processing modes across species. We also showed that PARE could provide independent supporting evidence during miRNA annotation when combined with known miRNA prediction/annotation tools, which are largely relied on small RNA sequencing (sRNA-seq) data.

## Materials and Methods

### Data Sources

PARE and sRNA-seq datasets from *Arabidopsis thaliana*, *Zea mays*, *Oryza sativa*, *Glycine max*, and *Solanum lycopersicum* were downloaded from the Sequence Read Archive (SRA) database (Leinonen et al., 2011). The accession numbers are listed in Supplementary Table 1. The SPARE datasets of *Arabidopsis thaliana* used in this study are stored under the accession numbers SRP021538 and SRP137005.

The miRNA annotation information for Arabidopsis, maize, and rice were retrieved from miRbase v.22 (Kozomara and Griffiths-Jones, 2011). The miRNA annotation information for soybean and tomato were obtained from the Plant small RNA genes website (Lunardon et al., 2020). The genome sequences of different species were retrieved from TAIR (TAIR10) (Lamesch et al., 2012), Ensembl Plants (B73_RefGen_v4, IRGSP-1.0, SL2.50) (Bolser et al., 2016), and the Plant small RNA genes (Glycine_max_V1-0) (Lunardon et al., 2020).

### Parallel Amplification of RNA Ends, Specific Parallel Amplification of RNA Ends, and sRNA-Seq Analysis

Fastq-dump was used to convert SRA format to fastq format (Leinonen et al., 2011). FastQC^1^ and fastp (Chen et al., 2018) were used for quality evaluation and adapter trimming, respectively. Reads with 20 nt (PARE), 18–51 nt (SPARE) and 17–26 nt (sRNA) were kept for further analysis. ShortStack (Axtell, 2013) was used to assign multiple-mapped PARE and sRNA-seq reads to the genome. Bowtie (Langmead, 2010) was used to map SPARE reads to the genome.

### miR-3p Information

For miR-3p with poor or no annotation, after loading merged sRNA-seq alignment bam files (Supplementary Table 1) with Integrative Genomics Viewer (IGV)-sRNA genome browser, we folded the precursor using the built-in RNAfold program and determined the miR-5p/miR-3p positions according to the most abundant reads on one side (usually the miR-5p in this case) and deduced the other side by the 2 nt 3′ overhang rule.

### miRNA Processing Modes Characterization

**FIGURE 1:**
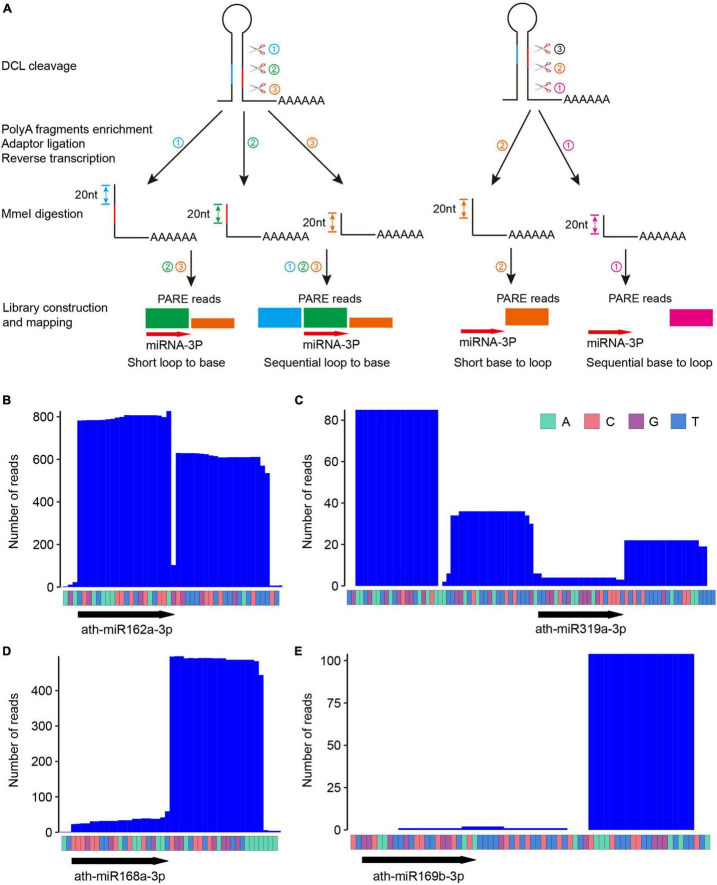
Parallel Amplification of RNA Ends (PARE) patterns reflect different miRNA processing modes. **(A)** Schematic diagram of PARE library construction and typical PARE patterns for different miRNA processing modes. ➀➁➂ depicts the order of cleavage. Note that for short loop-to-base processing, only two cuts (i.e., ➁➂) are executed, and for sequential processing, additional cuts (i.e., more than three cuts) may occur but are not shown here. Different colors of PARE signals reflects their sources of cleavage remnants. Bottom arrow highlights the position of miR-3p. **(B–E)** Degradome profiles of four selected miRNAs with known processing modes. ath-miR162a **(B)**, short loop to base; ath-miR319a **(C)**, sequential loop to base; ath-miR168a **(D)**, short base to loop; ath-miR169b **(E)**, sequential base to loop. Raw counts from four representative datasets with high abundance at respective analyzed miRNA locus (miR162a, SRR1171803; miR319a, SRR7652709; miR168a, SRR1171802, ath-miR169b, SRR7652709) were used for plotting the figures.

Only precursors with 10 reads in at least one sample, or five reads in at least two samples within a ±50 nt window surrounding miR-3p were kept. Deduction of miRNA processing modes from patterns of PARE signatures is depicted in Figure 1A and is determined manually.

## Results

### Deducing miRNA Processing Modes From Parallel Amplification of RNA Ends Patterns Around miR-3p

In PARE, polyadenylated RNAs are isolated, and an RNA adaptor containing an *Mme*I enzyme recognition site is ligated to the 5′ end of uncapped RNA fragments. *Mme*I cuts reverse transcribed DNA ∼20 nt downstream of its recognition site. The cleaved 20-nt tags are cloned and sequenced to track the 5′ end of uncapped RNAs (German et al., 2008). In principle, for miRNA processed from loop to base, two (short loop to base) or more (sequential loop to base) 3′ remnants of DCL cleavage products will be cloned. Meanwhile, for miRNA processed from base to loop, only one 3′ remnant of the DCL processing products downstream of the first cleavage site carries the poly(A) tail, and consequently will be cloned. Figure 1A shows schematic PARE patterns for different processing modes.

To test whether PARE is suitable for the characterization of miRNA processing modes, we collected 24 Arabidopsis PARE datasets from public databases (Supplementary Table 1). After removing adapters, the 20-nt of degradome tags were kept for further analysis. ShortStack software was employed to allocate multi-mapping reads based on the number of unique mapped reads on different loci (Johnson et al., 2016).

We selected four miRNAs with different processing modes pre-determined by SPARE and checked whether they displayed expected PARE patterns. For the short loop-to-base processed miR162a, we detected a major PARE peak at miR162a-3p and a minor peak afterward (Figure 1B). For the sequentially loop-to-base processed miR319a, two additional PARE peaks upstream of miR319a-3p were found (Figure 1C). For the short base-to-loop processed miR168a, one major peak right after miR168a-3p was observed (Figure 1D), and finally, for the sequentially base-to-loop processed miR169b, a distinct peak 21 nt downstream of miR169b-3p was detected (Figure 1E). These data indicate the robustness of PARE in miRNA processing mechanism recognition.

### Quality Control for miRNAs Contamination

**FIGURE 2:**
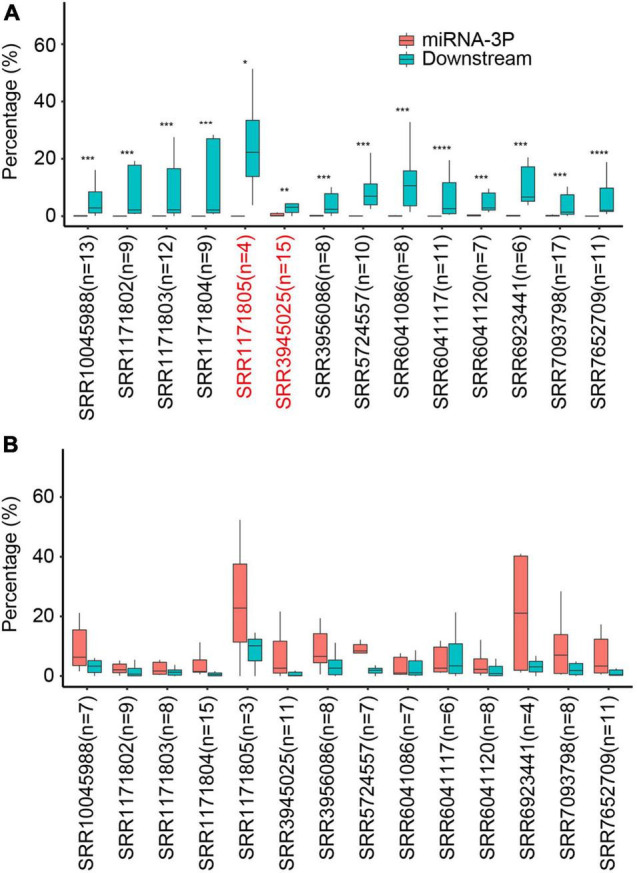
Quality control of miRNA contamination of Arabidopsis Wt Parallel Amplification of RNA Ends (PARE) libraries. **(A,B)** Relative abundance of miR-3p and downstream PARE reads from known short base-to-loop processed miRNAs **(A)** or short loop-to-base processed miRNAs **(B)** pre-determined by Specific Parallel Amplification of RNA Ends (SPARE). Percentage was calculated as reads of miR-3p tags or downstream tags vs. total reads from miR-3p and downstream tags of miRNAs with respective processing modes. For each PARE library, only miRNAs with a percentage of miR-3p or downstream tags >0.5%, and a sum number >10 were kept. The number of analyzed miRNAs in each library are indicated in brackets. *p < 0.05; **p < 0.01; ***p < 0.005, ****p < 0.001 (Wilcoxon test). SRR6923441, SRR6923442, and SRR6923443 were merged under the entry of SRR6923441, owing to low sequencing depth.

Because only 20-nt degradome tags were retrieved after *Mme*I digestion, one major concern regarding the use of PARE in characterizing miRNA processing modes was the contamination of mature miRNAs (Lu et al., 2009). As such, a quality control step for each PARE dataset was crucial to avoid misidentification. As described above, base-to-loop processed miRNAs should have no miR-3p tags if there is no contamination; this could therefore be used to estimate the miRNAs contamination levels of PARE data. 16 wild-type (Wt) PARE libraries (Three of them from a same experiment were combined due to low sequencing depth) were analyzed for possible miRNA contamination. Remarkably, for miRNAs with known short base-to-loop processing modes, distinct PARE tags were observed right after miR-3ps, with almost no miR-3ps detected in most analyzed libraries (Figure 2A). In sharp contrast, for miRNAs with known short loop-to-base processing modes, significantly higher miR-3p tags than downstream tags were detected (Figure 2B). This strongly suggested that in most PARE libraries the contamination of mature miRNAs was low and had negligible impact on miRNA processing modes determination. Twelve out of 14 libraries passed the quality control step with a stringent *p*-value cut-off (0.005).

### Parallel Amplification of RNA Ends as a Promising Tool in Systematic Characterization of miRNA Processing Modes

**FIGURE 3:**
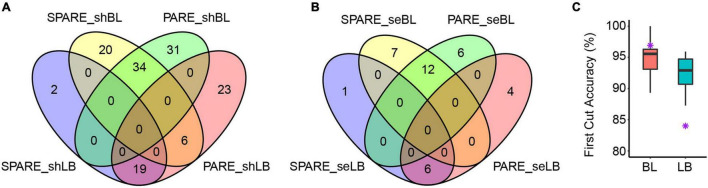
Comparison of Parallel Amplification of RNA Ends (PARE) and Specific Parallel Amplification of RNA Ends (SPARE) results in Arabidopsis. **(A)** Venn diagram showing the number of short base-to-loop and short loop-to-base processed miRNAs as determined by PARE and SPARE. **(B)** Venn diagram showing the number of sequential base-to-loop and sequential loop-to-base processed miRNAs as determined by PARE and SPARE. shBL, short base to loop; shLB, short loop to base; seBL, sequential base to loop; seLB, sequential loop to base. **(C)** First cut accuracy of PARE in miRNA processing. Purple star, first-cut accuracy of SPARE (SRR6930534). LB, loop to base; BL, base to loop. Calculation of first cut accuracy was performed according to Moro et al. (2018).

We next determined the miRNA processing modes in Arabidopsis based on PARE patterns and compared them with SPARE results. We not only validated most of the results (71/107, 66%) by SPARE, but, more importantly, we determined the processing modes of 36 additional miRNAs (Supplementary Table 2 and Figures 3A,B). In particular, PARE showed comparable first-cut accuracy as SPARE (Figure 3C; Moro et al., 2018), indicating the robustness of PARE in the characterization of miRNA processing modes. The processing modes of 49 miRNAs from the two unpassed libraries were identified, and surprisingly all were concordant with those from passed libraries (Supplementary Figure 1).

**FIGURE 4:**
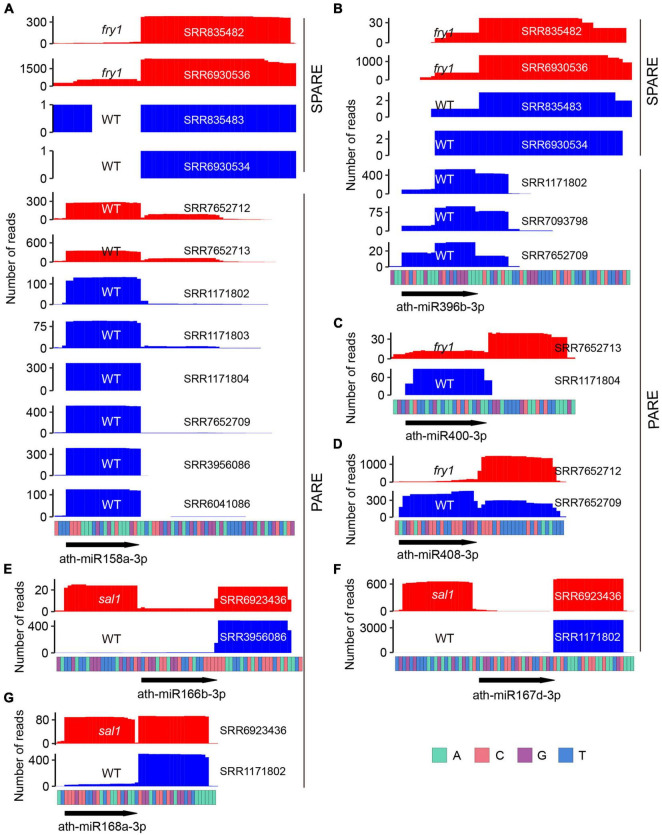
*fry1*/*sal1* impacts miRNA processing. **(A)** Specific Parallel Amplification of RNA Ends (SPARE) and Parallel Amplification of RNA Ends (PARE) profiles of ath-miR158a-3p in *fry1* (red) and Wt (blue). **(B)** SPARE and PARE profiles of ath-miR396b-3p in *fry1* (red) and Wt (blue). **(C,D)** PARE profiles show that the processing modes of ath-miR400 **(C)** and ath-miR408 **(D)** changed from short loop-to-base to short base-to-loop in *fry1*. **(E–G)** PARE profiles show that the processing modes of ath-miR166b **(E)**, ath-miR167d **(F)**, and ath-miR168a **(G)** changed from short base-to-loop to short/sequential loop-base in *sal1*.

Notably, six miRNAs (miR158a, miR390a, miR390b, miR391, miR396a, and miR396b) displayed inconsistent processing modes between PARE and SPARE (Figure 3A). For miR391, the conclusion by PARE may have been inaccurate as only a few degradome tags were obtained (Supplementary Figure 2). For the remaining five miRNAs, SPARE annotated them as short base-to-loop, but all PARE data treated them as short loop-to-base. We thus reanalyzed the SPARE signals around these miRNAs. For miR158a, miR390b, miR396a, and miR396b, robust signals were only detected in the *fiery1* (*fry1*) mutant but not in the Wt (Figures 4A,B and Supplementary Figures 3B,C). For miR390a, robust and consistent signals were detected in both *fry1* and Wt (Supplementary Figure 3A). *fry1* has been frequently used in PARE and SPARE experiments because the *FRY1* mutation attenuates RNA degradation from 5′ to 3′ by XRNs, thereby accumulating more miRNA targets cleavage remnants and miRNA processing intermediates (Gy et al., 2007). A comparison between Wt and *fry1* revealed additional inconsistent patterns, including miR400 and miR408 (Figures 4C,D). More strikingly, in another amino acid substitution allele of FRY1, *sal1/alx8* (Wilson et al., 2009), the processing of more miRNAs changed from short base-to-loop to short/sequential loop-to-base, including miR166b, miR167d, miR168a, miR403, miR841a, miR841b, and miR850 (Figures 4E–G and Supplementary Figures 3D–G), implying that the SAL1/FRY1 mutation may influence the characterization of miRNA processing and should be used carefully.

### Identification of miRNA Processing Modes in Four Crops

**FIGURE 5:**
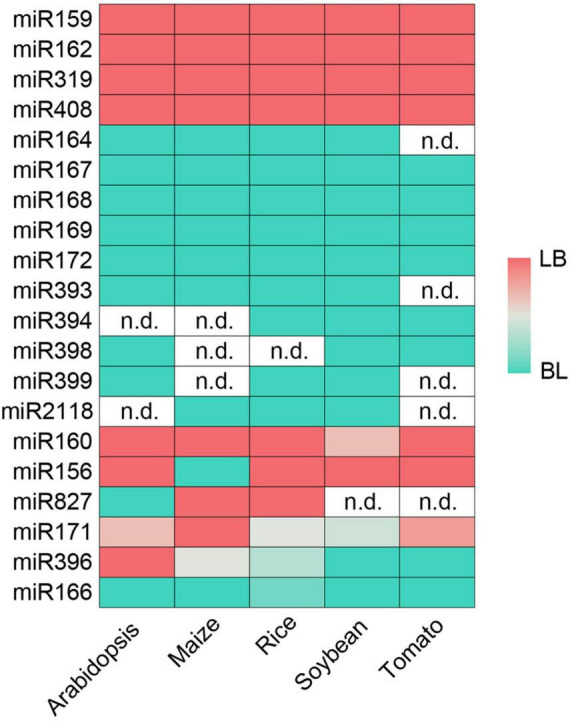
Conservation of miRNA processing modes determined by Parallel Amplification of RNA Ends (PARE). LB, short/sequential loop to base; BL, short/sequential base to loop. The intensity of color reflects the degree of intraspecific conservation. White (n.d.): not determined.

Though SPARE results from crops are lacking, evolutionary conservation analysis reveals that miRNAs within a same family tend to share conserved processing modes across angiosperms (Chorostecki et al., 2017). We retrieved 6, 16 (Four of them from a same experiment were combined due to low sequencing depth), 14, and 13 PARE datasets of *Zea mays*, *Oryza sativa*, *Glycine max* and *Solanum lycopersicum* from public databases (Supplementary Table 1). MiRNAs whose counterparts in Arabidopsis are processed in short base-to-loop were used to evaluate the miRNA contamination levels in each library (Supplementary Figure 4). After filtering with a *p*-value cut-off of 0.05, two, nine, four, and seven libraries from *Zea mays*, *Oryza sativa*, *Glycine max* and *Solanum lycopersicum*, respectively, were kept for further analysis. For miRNAs lacking miR-3p annotation, sRNA-seq data (Supplementary Table 1) and RNA folding structures were used to infer their positions based on the 2-nt overhang rule of the miR-5p/miR-3p duplexes (Supplementary Table 3). Following the procedures described above, we obtained the degradome profiles around miR-3ps. We determined the processing modes of 36, 91, 90, and 54 miRNAs in *Zea mays*, *Oryza sativa*, *Glycine max*, and *Solanum lycopersicum*, respectively (Supplementary Table 2). The relatively fewer numbers in crops compared with Arabidopsis are likely owing to poor annotation of miRNAs and low or even no expression because of the limited amount of sequencing data. Consistent with previous notions (Chorostecki et al., 2017), an inspection of processing modes identified in at least three species revealed that 14 miRNA families (i.e., miR159, miR162, miR164, miR167, miR168, miR169, miR172, miR319, miR393, miR394, miR398, miR399, miR408, and miR2118) shared conserved processing modes (Figure 5). Members of the miR171 family in Arabidopsis utilize different processing modes (Bologna et al., 2013). Here, we also detected different processing modes in five miRNA families (i.e., miR156, miR160, miR166, miR396, and miR827) at different degrees (Figure 5). These analyses suggest that although members in the same miRNA family tend to share the same processing mode, differential processing modes may occur at intraspecific or interspecific levels.

### Parallel Amplification of RNA Ends Assists miRNA Annotation

**FIGURE 6:**
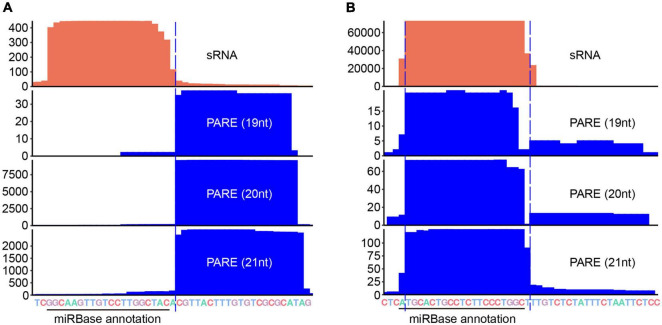
Parallel Amplification of RNA Ends (PARE) corrects miR-3p annotations. Coverage of sRNA (orange) and PARE (blue) read alignments around ath-miR169a-3p **(A)** and gma-miR408c-3p **(B)**. Black solid line, miRBase annotated miR-3p border; blue dashed line, PARE corrected miR-3p border. Merged sRNA datasets (Supplementary Table 1) were used for sRNA analysis and datasets under the accession numbers SRR1171802 (for ath-miR169a-3p) and SRR1451679 (for gma-miR408c-3p) were used for PARE analysis.

Accurate detection of miRNA processing remnants suggests that PARE is a useful tool for assisting miRNA annotation and/or prediction. We re-examined the annotation of miRBase and corrected 43 records (Supplementary Table 4). For instances, the Arabidopsis miR169 family has 14 members with miR169a being the most abundant (Bologna et al., 2013). According to the annotation from miRBase, the length of ath-miR169a-3p is 20-nt with the sequence being GGCAAGUUGUCCUUGGCUAC. The 3′ end of the ath-miR169a-3p reads from sRNA-seq data is ragged. In sharp contrast, we detected a sharp degradome signal starting 1 nt downstream of the end of the miRBase annotation (Figure 6A), revealing that the correct ath-miR169a-3p sequence should be 21-nt in size (i.e., GGCAAGUUGUCCUUGGCUACA). Gma-miR408c belongs to the conserved miR408 family and is processed in a short loop-to-base direction. The miRBase annotation shows that the sequence of gma-miR408c-3p is AUGCACUGCCUCUUCCCUGGC. We detected a major degradome peak that began 1 nt downstream of the above annotation (Figure 6B), suggesting that the majority of Gma-miR408c-3p started 1 nt after the annotated start site (i.e., UGCACUGCCUCUUCCCUGGCU). In both cases, the corrected version but not the miRBase annotation meets the 2-nt overhang rule (Supplementary Figure 5). We also predicted the targets of different versions of ath-miR169a-3p and gma-miR408c-3p with TarHunter and determined their cleavage site using PARE datasets (Ma et al., 2017) (Supplementary Figure 6 and Supplementary Table 5). For ath-miR169a-3p, one same potential target was retrieved, which showed a weak target plot (T-plot) signal. This could be due to the fact that ath-miR169a-3p is the passenger strand of the miR169a/* duplex. For gma-miR408c-3p, multiple conserved copper-related targets were identified with strong T-plots signals for both versions (Ma et al., 2015). An additional target (EBP1) was retrieved only for the corrected version (Supplementary Figure 6 and Supplementary Table 5). Importantly, all the cleavage sites were located at positions complementary to the 10th and the 11th nucleotides of the corrected gma-miR408c-3p, which is canonical for miRNA-guided slicing (Kasschau et al., 2003).

Taken together, our study suggested that PARE was effective for comprehensively analyzing miRNA processing modes and assisting miRNA annotation in an untargeted manner with satisfactory accuracy.

## Discussion

Having more variable hairpin sizes and fold-back structures than their animal counterparts, plants evolve more complicated miRNA processing modes. Hitherto, systematic investigation of miRNA processing modes has only been conducted in the model plant Arabidopsis using SPARE. Though the same miRNA families across different species tend to share conserved processing modes, exceptions are frequently observed, and as such, experimental approaches for the characterization of miRNA processing modes are indispensable.

Although effective, SPARE is a targeted approach that only captures selected DCL processing remnants and is time-consuming. By contrast, PARE is an untargeted approach that captures 5′ end uncapped and 3′ end polyadenylated RNAs. More importantly, PARE is commercialized and has been widely used to determine the miRNA targets in various plant species and many PARE datasets are publicly available. In principle, PARE is capable of tracking miRNA processing intermediates and has been used to determine the processing modes of miR319 and miR159 (Addo-Quaye et al., 2009; Li et al., 2011). Yet, concerns of potential miRNA contamination impede its application in the systematic characterization of miRNA processing modes (Lu et al., 2009). Here, we provide a solution for evaluating miRNA contamination and demonstrate that PARE can be used to dissect miRNA processing modes globally. As an untargeted approach, PARE can identify miRNA processing modes in an unbiased manner. On the other hand, PARE has less sensitivity and requires higher sequencing depth. Moreover, some PARE libraries may have higher miRNA contamination that influence the prediction (Figure 2 and Supplementary Figure 4). Thus, a quality control step for miRNA contamination is crucial for accuracy. Alternatively, replacing *Mme*I with *Eco*P15I, which produces 27-nt tags will effectively overcome this defect (Addo-Quaye et al., 2009; Li et al., 2019). Overall, SPARE and PARE can complement each other in dissecting miRNA processing modes in plants.

During our analysis, we also frequently detected obvious degradome signals at the beginning of the miR-5p or internal of miRNAs (Supplementary Figure 7). This could be owing to partial or misprocessing by DCL1, or slicing by ARGONAUTE 1 (AGO1) (Bologna et al., 2013). Other possible causes include: (i) 5′ processing remnants are re-polyadenylated and captured during library construction (Chekanova et al., 2007); (ii) the 5′ end of 3′ processing remnants are trimmed *in vivo* or during library construction *in vitro*; and (iii), multiple overlapped miRNAs/siRNAs exist in the same primary transcript that generates complex processing remnants (Reinhart et al., 2002; Allen et al., 2004; German et al., 2008).

*FRY1/SAL1* encodes a phosphatase with dual activities, including converting 3′-phosphoadenosine 5′-phosphosulfate (PAPS) into adenosyl phosphosulfate (APS) and dephosphorylates 3′-phosphoadenosine 5′-phosphate (PAP) to adenosine 5′-phosphate (AMP) (Quintero et al., 1996; Gil-Mascarell et al., 1999). *FRY1* plays important roles in multiple biological processes including post-transcriptional gene silencing (PTGS) (Gy et al., 2007) and RNA quality control (You et al., 2019). In the *fry1* mutant, the accumulation of toxic PAP in *fry1* impaired the activity of 5′ to 3′ exoribonucleases (XRNs). Consequently, *MIRNA*-derived loop and 3′ products become over-accumulated owing to the inhibition activities of XRN2 and XRN3 (Gy et al., 2007). It has been reported that 90% of degradome tags of loop-to-base processed miRNAs in *fry1* correspond to the position of the second cleavage site (Moro et al., 2018), indicating that *FRY1* may influence miRNA processing. Strikingly, opposite processing modes were frequently detected between Wt and *fry1*/*sal1* (Figure 4 and Supplementary Figure 3). Moreover, dramatic elevated partial cleavage was observed only in *fry1/sal1* (Supplementary Figure 8). Thus, *fry1* should be used only cautiously in the identification of processing mode. We analyzed the *fry1* small RNA sequencing data and found that misprocessed miRNA ratios were slightly elevated in *fry1-6* and *fry1-8* (Supplementary Figure 9), suggesting that FRY1 may also affect DCL1 processing. Nuclear exosome components HUA ENHANCER 2 (HEN2) and SUPPRESSOR OF PAS2 1 (SOP1) act on miRNA precursor degradation with selective impacts on loop-to-base miRNA processing when HYPONASTIC LEAVES 1 (HYL1) is impaired (Gao et al., 2020). It will be interesting to investigate the relationship between 5′ to 3′ and 3′ to 5′ degradation pathways on miRNA processing.

In plants, exhaustive efforts have been paid to miRNA prediction and annotation with a huge number of sRNA-seq datasets and different prediction tools. Although miRNA isoforms are frequently reported, the heterogenous ends of sRNA reads from sRNA-seq data can lead to misannotation, which may cause mistaken inferences about their AGO sorting and/or target identification (Mi et al., 2008; Zhang et al., 2014; Supplementary Figure 6). As an independent method, we showed that PARE can assist with accurate miRNA annotation (Supplementary Table 4 and Figure 6). Moreover, owing to the complexity of small RNA compositions in plants, false positives lead to many questionable miRNA annotations, which have now become a major concern to the community (Axtell and Meyers, 2018). To solve this issue, the Axtell group developed ShortStack, which has high precision and near-zero false positives; however, it shows limited sensitivity and high false negatives. By analyzing 28 Arabidopsis sRNA-seq libraries, only 143 out of 325 entries in miRBase were designated as *bona fide* miRNAs by ShortStack (Lunardon et al., 2020). Unpassed entries were largely due to imprecise processing, unpaired bases, bulges limitation and undetected miRNA*. We suggest that PARE may also be used to help with miRNA characterization by tracking processing remnants. Our preliminary analysis revealed that 113 entries had robust PARE support, including 30 miRNAs that were not designated as miRNAs by ShortStack (Supplementary Figure 10). We believe that integration of degradome signatures into miRNA prediction tools will improve both accuracy and sensitivity.

In conclusion, we provided a solution for the estimation of miRNA contamination and demonstrated the capacity of PARE in characterizing miRNA processing and miRNA annotation.

## Data Availability Statement

The original contributions presented in the study are included in the article/Supplementary Material, further inquiries can be directed to the corresponding author.

## Author Contributions

GR and NL conceived the idea and wrote the manuscript. NL conducted the analysis. Both authors contributed to the article and approved the submitted version.

## Conflict of Interest

The authors declare that the research was conducted in the absence of any commercial or financial relationships that could be construed as a potential conflict of interest.

## Publisher’s Note

All claims expressed in this article are solely those of the authors and do not necessarily represent those of their affiliated organizations, or those of the publisher, the editors and the reviewers. Any product that may be evaluated in this article, or claim that may be made by its manufacturer, is not guaranteed or endorsed by the publisher.

## Footnotes

1
https://www.bioinformatics.babraham.ac.uk/projects/fastqc/
