# Supplementary material for: Systematic Characterization of MicroRNA Processing Modes in Plants With Parallel Amplification of RNA Ends
Source: Front Plant Sci. 2021 Dec 7;12:793549. doi: 10.3389/fpls.2021.793549 (PMC8688358; doi:10.3389/fpls.2021.793549)
Supplement: Supplementary file 1 [file Data_Sheet_1.PDF]

Supplemental information

Systematic characterization of microRNA processing modes in plants with Parallel Amplification of RNA Ends (PARE)

Ning Li[1], Guodong Ren[1,*]

1, State Key Laboratory of Genetic Engineering and Ministry of Education Key Laboratory for Biodiversity Science and Ecological Engineering, Institute of Plant Biology, School of Life Sciences, Fudan University, Shanghai 200438, China

***: Corresponding author:**

Guodong Ren

Tel: 86-21-3124-6776

E-mail: gdren@fudan.edu.cn

Supplemental Figures and Legends

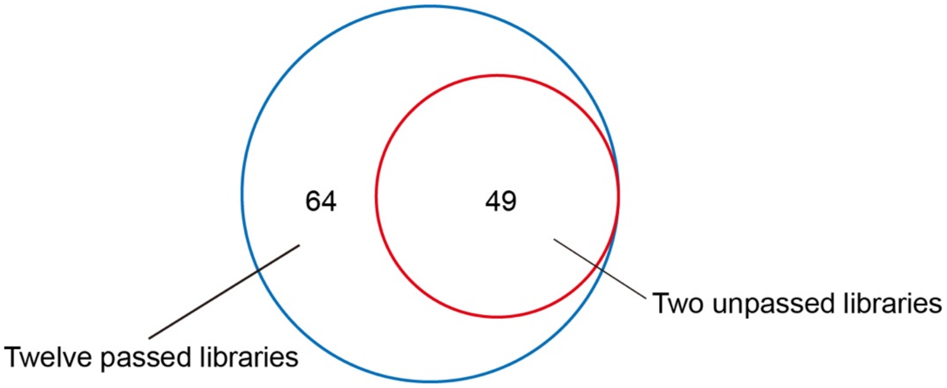

Supplemental Figure 1. Venn diagram showing the number and consistency of miRNAs processing modes determined from the twelve passed libraries and two unpassed libraries.

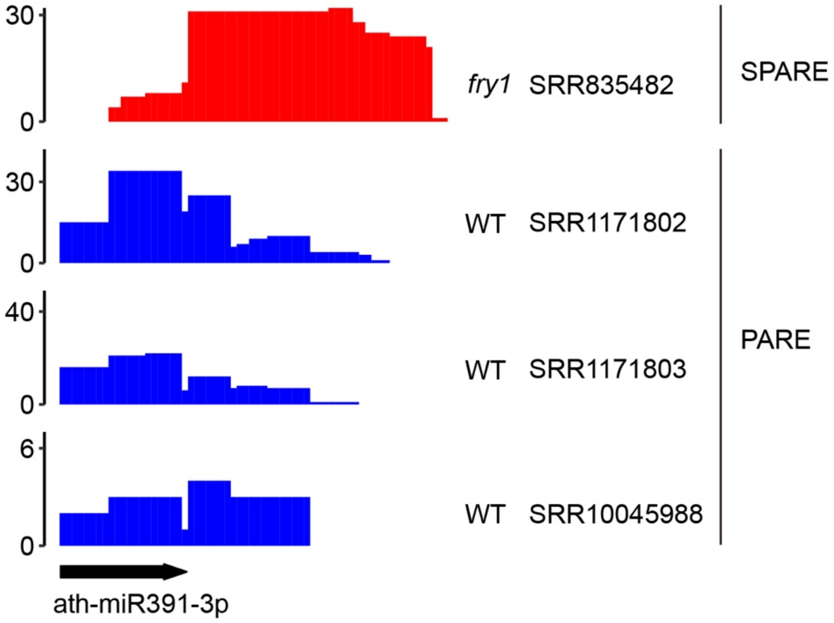

Supplemental Figure 2. SPARE and PARE profiles of ath-miR391-3p in *fry1* (red) and Wt (blue).

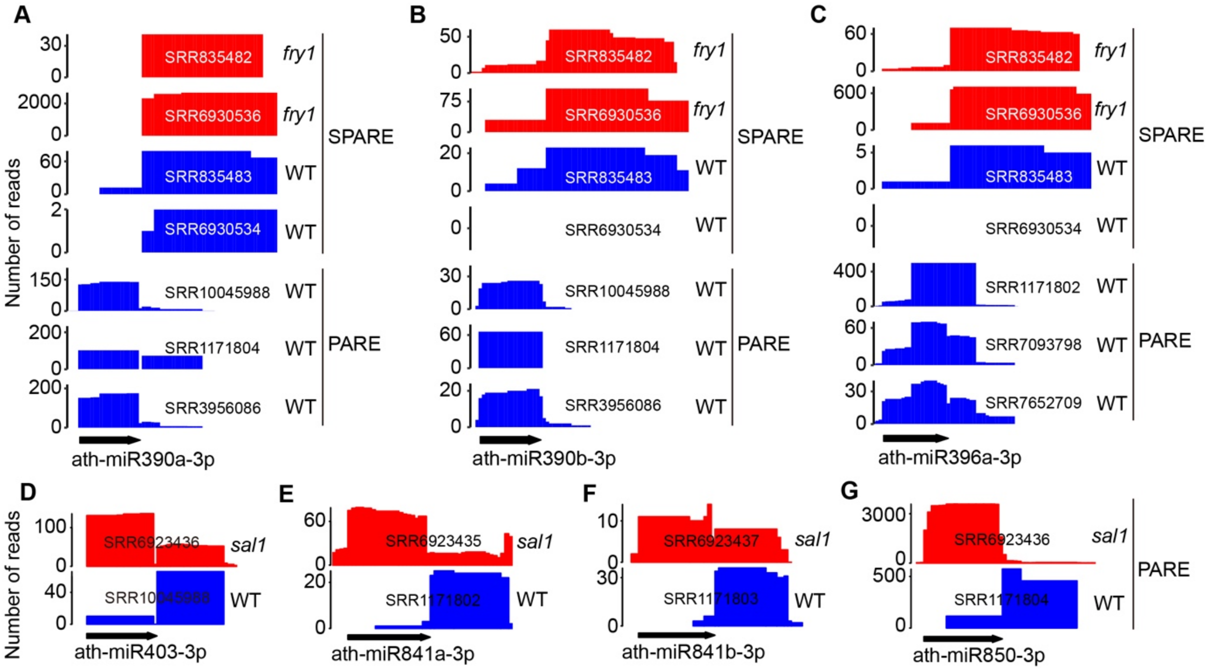

Supplemental Figure 3. *fry1*/*sal1* impacts miRNA processing. **(A-C)** SPARE and PARE profiles of ath-miR390a-3p **(A)**, ath-miR390b-3p **(B)** and ath-miR396a-3p **(C)** in *fry1* (red) and Wt (blue). **(D-G)** PARE profiles show that the processing modes of miR403 **(D)**, miR841a **(E)**, miR841b **(F)** and miR850 **(G)** change from short base-to-loop to short loop-base in *sal1*.

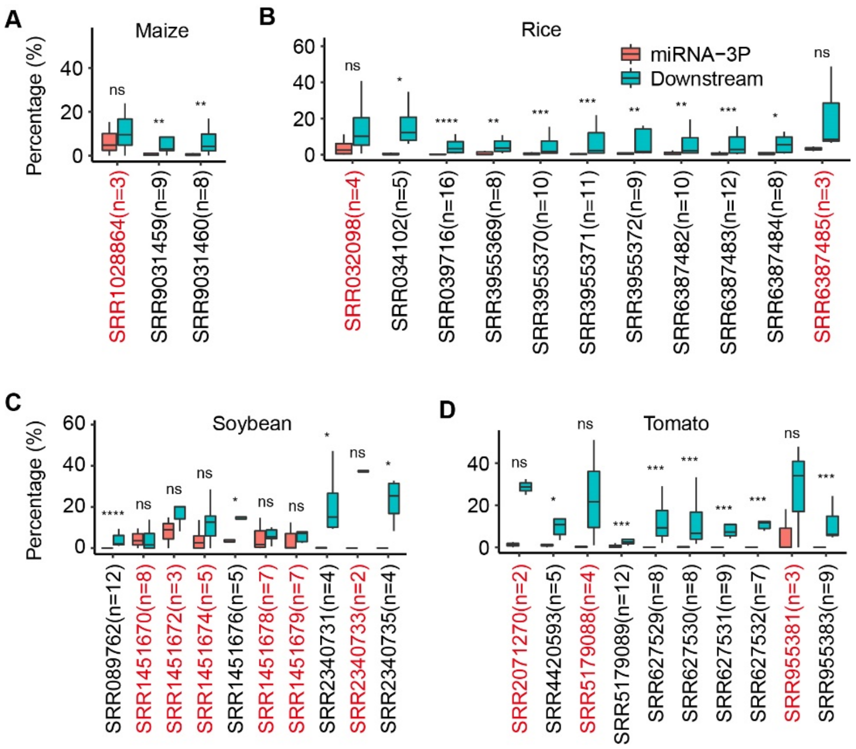

Supplemental Figure 4. Quality control of miRNA contamination. **(A-D)** shows relative

abundance of miR-3p and downstream PARE reads from short base-to-loop processed miRNAs (based on likely family-level conservation) of maize **(A)**, rice **(B)**, soybean **(C)** and tomato **(D)**. Percentage was calculated as reads of miR-3p tags or downstream tags vs total reads from miR-3p and downstream tags of miRNAs with respective processing modes. For each PARE library, only miRNAs with a percentage of miR-3p or downstream tags >0.5%, and a sum number >10 were kept. The number of analyzed miRNAs in each library are indicated in brackets. *, p-value <0.05; **, p-value <0.01; ***, p-value <0.005, ****, p-value <0.001 (Wilcoxon test). SRR039716, SRR039717, SRR039718, SRR039719 and SRR039720 were merged under the entry of SRR039716, due to low sequencing depth.

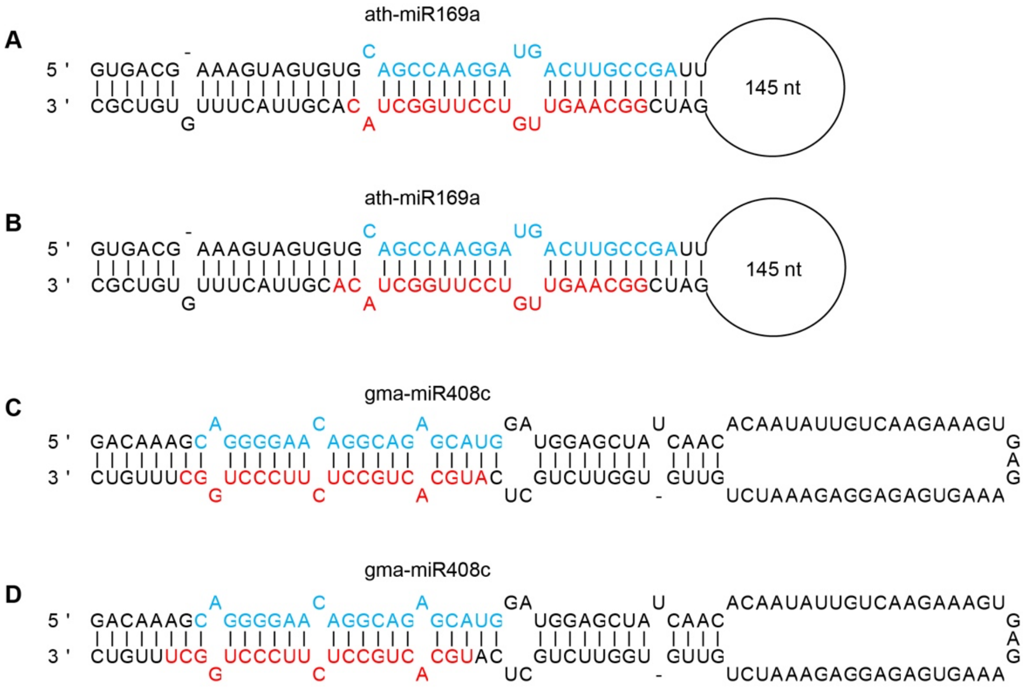

Supplemental Figure 5. PARE assists miR-3p annotation. **(A-B)** shows the positions of ath-miR169a-5p/-3p of the miRBase version (A) and the corrected version (B). **(C-D)** shows the positions of gma-miR408c-5p/3p of the miRBase version (C) and the corrected version (D). Blue, miR-5p; red, miR-3p.

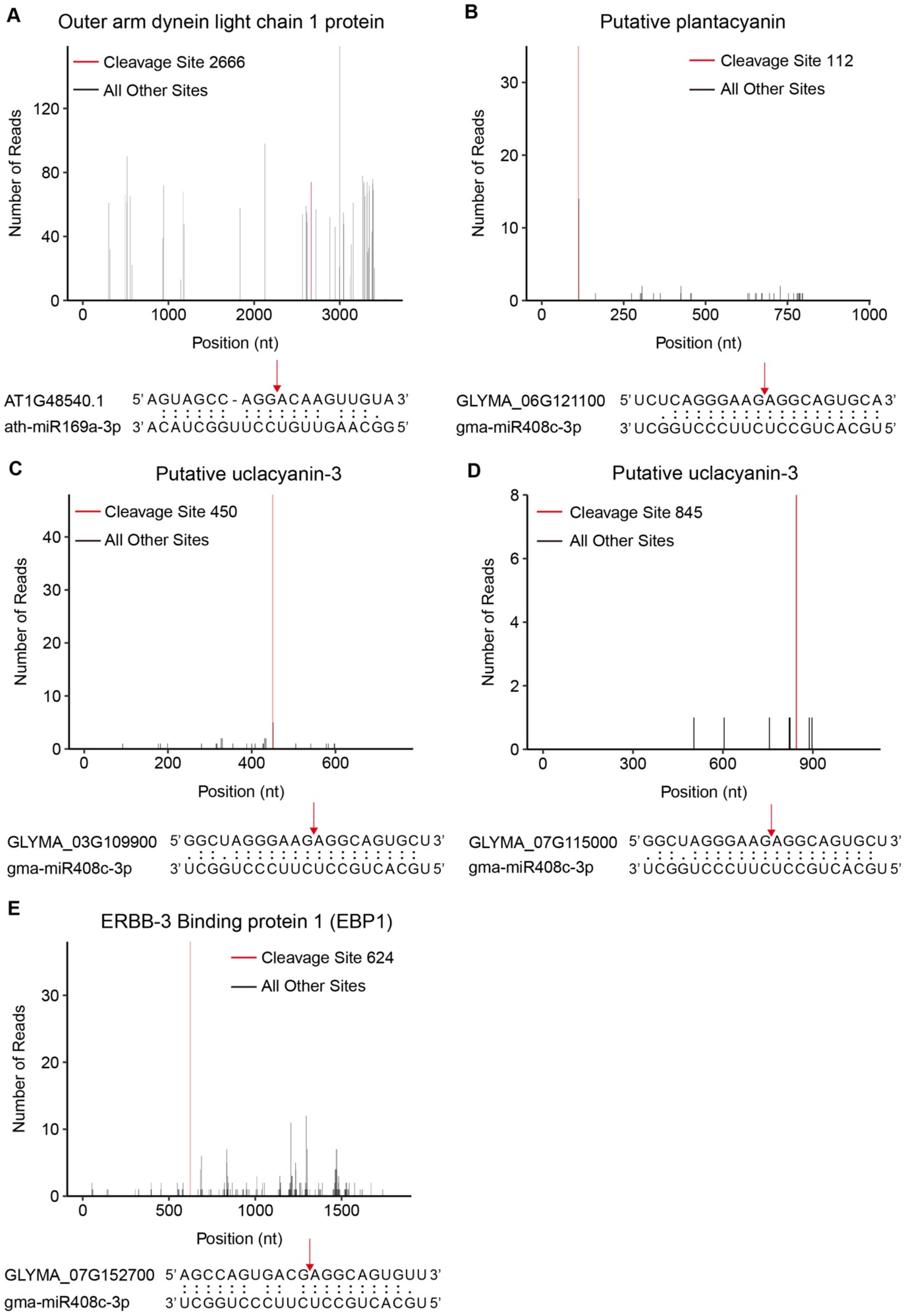

Supplemental Figure 6. T-plots showing the cleavage signals of ath-miR169a-3p and gma-miR408c-3p targets. Red lines and arrows indicate respective cleavage site. Datasets under the

accession numbers SRR1171804 (for ath-miR169a-3p target) and GSE58779 (for gma-miR408c-3p targets) were used for analysis.

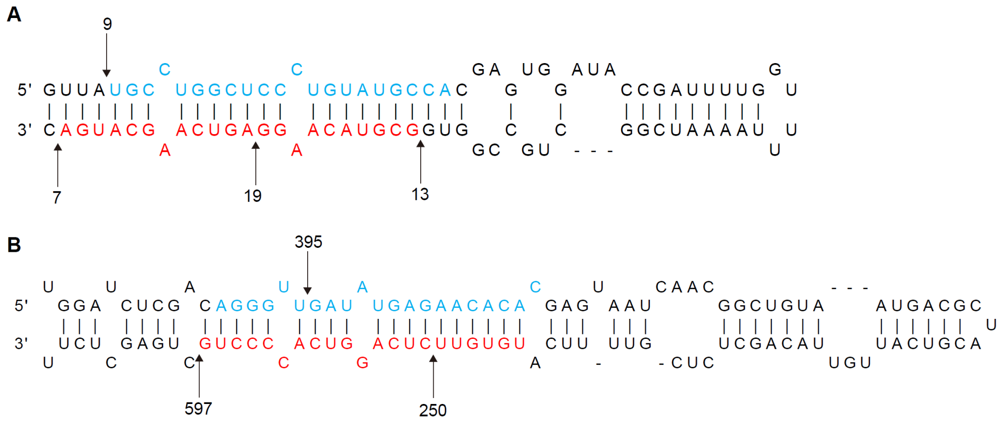

Supplemental Figure 7. Misprocessing of Arabidopsis miRNA precursors. (A-B) show predicted secondary structure of *ath-MIR160c* (A) and *ath-MIR398b* (B), and the number processing intermediates detected by PARE. The miR-5p and miR-3p sequences are highlighted in blue and red, respectively. Datasets under the accession numbers SRR7093798 (for ath-MIR160c) and SRR1171805 (for ath-miR398b) were used for analysis.

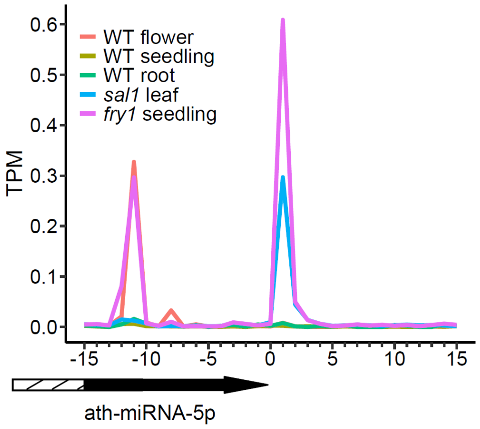

Supplemental Figure 8. Partial cleavage intermediates elevates in *fry1*/*sal1*. Position 0 corresponds to the 3' end of miR-5p.

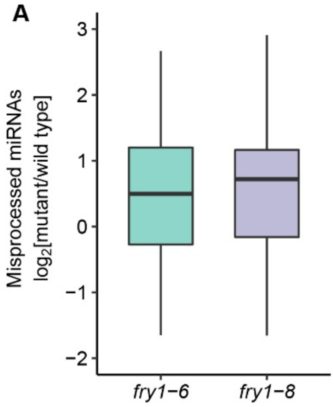

Supplemental Figure 9. miRNA processing imprecision in *fry1* mutants. Dataset under the accession number GSE126482 was used for analysis. MiRNA processing accuracy was performed according to (Liu et al., 2012).

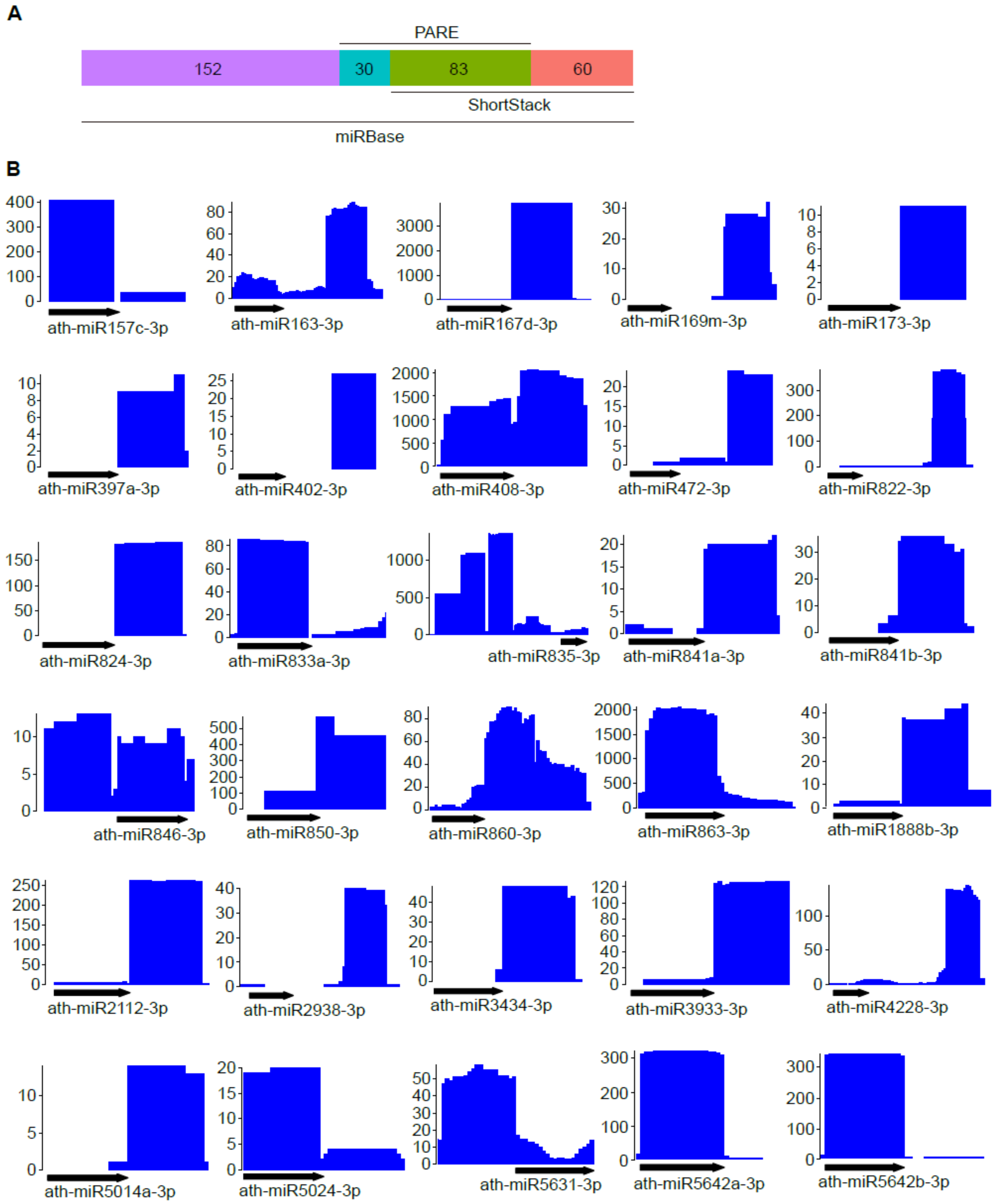

Supplemental Figure 10. PARE signals assist miRNA annotation. **(A)** Bar plot showing the overlaps among the number of miRNAs in miRBase, validated by ShortStack and having independent supporting signals by PARE. **(B)** Representative PARE profiles of 30 miR-3ps detected by PARE but not ShortStack.

Supplemental Tables

Supplemental Table 1. PARE and sRNA-seq data information retrieved from public database.

Supplemental Table 2. Summary of miRNA processing modes in Arabidopsis, maize, rice, soybean, and tomato.

Supplemental Table 3. miR-3p positions determined by sRNA-seq and RNA folding.

Supplemental Table 4. Revised miR-3p positions by PARE.

Supplemental Table 5. Predicted targets of ath-miR169a-3p and gma-miR408c-3p.

References:

Liu, C., Axtell, M.J., and Fedoroff, N.V. (2012). The helicase and RNaseIIIa domains of Arabidopsis Dicer-Like1 modulate catalytic parameters during microRNA biogenesis. *Plant Physiol* 159(2), 748-758. doi: 10.1104/pp.112.193508.
